# Supplementary material for: Treatment and Outcomes of Clostridioides difficile Infection in Switzerland: A Two-Center Retrospective Cohort Study
Source: J Clin Med. 2022 Jun 30;11(13):3805. doi: 10.3390/jcm11133805 (PMC9267637; doi:10.3390/jcm11133805)
Supplement: Supplementary file 1 [file jcm-11-03805-s001.zip › jcm-1784237-supplementary.pdf]

Table S1. Evolution of diagnostic tests over time in Lausanne

Characteristic	2014<br>N = 113[1]	2015<br>N = 74[1]	2016<br>N = 64[1]	2017<br>N = 102[1]	2018<br>N = 67[1]	Overall,<br>N = 420[1]
NAAT	4.4% [5]	2.7% [2]	67.2% [43]	15.7% [16]	100.0% [67]	31.7% [133]
GDH	95.6% [108]	100.0% [74]	100.0% [64]	81.4% [83]	92.5% [62]	93.1% [391]
Toxin A/B EIA	40.7% [46]	86.5% [64]	75% [48]	54.9% [56]	62.7% [42]	61.0% [256]
Toxigenic culture	32.7% [37]	0.0% [0]	10.9% [7]	42.2% [43]	0.0% [0]	20.7% [87]
Ribotype 027	0.0% [0]	0.0% [0]	0.0% [0]	2.0% [2]	0.0% [0]	0.5% [2]
Binary toxin positive	0.0% [0]	0.0% [0]	0.0% [0]	12.7% [13]	7.5% [5]	4.3% [18]

[1] % [n]

Table S2. Evolution of diagnostic tests over time in Zurich

Characteristic	2015<br>N = 91[1]	2016<br>N = 98[1]	2017<br>N = 136[1]	2018<br>N = 81[1]	Overall,<br>N = 406[1]
NAAT	98.9% [90]	100.0% [98]	100.0% [136]	98.8% [80]	99.5% [404]
GDH	98.9% [90]	99.0% [97]	100.0% [136]	100.0% [81]	99.5% [404]
Toxin A/B EIA	0.0% [0]	0.0% [0]	0.0% [0]	0.0% [0]	0.0% [0]
Toxigenic culture	0.0% [0]	0.0% [0]	0.0% [0]	0.0% [0]	0.0% [0]
Ribotype 027	0.0% [0]	0.0% [0]	0.0% [0]	0.0% [0]	0.0% [0]
Binary toxin positive	0.0% [0]	0.0% [0]	0.0% [0]	0.0% [0]	0.0% [0]

[1] % [n]

Table S3. Evolution of treatment over time and center

	2014	2015		2016		2017		2018		Overall	
Molecule	Lausanne (N = 60[1])	Lausanne (N = 67[1])	Zurich (N = 76[1])	Lausanne (N = 54[1])	Zurich (N = 86[1])	Lausanne (N = 91[1])	Zurich (N = 120[1])	Lausanne (N = 60[1])	Zurich (N = 62[1])	Lausanne (N = 332[1])	Zurich (N = 344[1])
Fidaxomicin	0.0% [0]	0.0% [0]	0.0% [0]	1.9% [1]	0.0% [0]	6.6% [6]	0.0% [0]	35.0% [21]	0.0% [0]	8.4% [28]	0.0% [0]
Metronidazole	86.7% [52]	83.6% [56]	93.4% [71]	83.3% [45]	95.3% [82]	70.3% [64]	96.7% [116]	40.0% [24]	90.3% [56]	72.6% [241]	94.5% [325]
Vancomycine	13.3% [8]	16.4% [11]	6.6% [5]	4.7% [4]	4.7% [4]	23.1% [21]	3.3% [4]	25.0% [15]	9.7% [6]	19.0% [63]	5.5% [19]

[1] % [n]

Table S4. Comparison of metronidazole and vancomycin for the composite outcome.

Characteristic	Negative score<br>N = 384[1]	Positive score (Death, no cure or recurrence)<br>N = 265[1]	Overall<br>N = 649[1]	p-value[2]
Molecule				>0.9
Fidaxomicin	NA% [0]	NA% [0]	NA% [0]	
Metronidazole	59.1% [333]	40.9% [230]	100.0% [433]	
Vancomycin	59.3% [51]	40.7% [35]	100.0% [86]	

[1] % [n]

[2] Fisher's exact test

Table S5. Comparison of metronidazole and vancomycin for recurrence at week 8.

Characteristic	No recurrence at 8 weeks<br>N = 418[1]	Recurrence at 8 weeks<br>N = 76[1]	Overall<br>N = 494[1]	p-value[2]
Molecule				0.13
Fidaxomicin	NA% [0]	NA% [0]	NA% [0]	
Metronidazole	83.6% [362]	16.4% [71]	100.0% [433]	
Vancomycin	91.8% [56]	8.2% [5]	100.0% [61]	

[1] % [n]

[2] Fisher's exact test

Table S6. Comparison of metronidazole and vancomycin for clinical failure.

Characteristic	Failure<br>N = 86[1]	Clinical cure<br>N = 562[1]	Overall<br>N = 648[1]	p-value[2]
Molecule				0.3
Fidaxomicin	NA% [0]	NA% [0]	NA% [0]	
Metronidazole	12.7% [72]	87.3% [494]	100.0% [566]	
Vancomycin	17.1% [14]	82.9 % [68]	100.0% [82]	

[1] % [n]

[2] Fisher's exact test
